# NcSWP8, a New Spore Wall Protein, Interacts with Polar Tube Proteins in the Parasitic Microsporidia *Vairimorpha (Nosema) ceranae*

**DOI:** 10.3390/microorganisms13010142

**Published:** 2025-01-12

**Authors:** Pengfei Wang, Dufu Li, Qianmin Hai, Siming Liu, Yueyue Zhang, Jun Zhang, Jinshan Xu, Zhengang Ma, Zeyang Zhou

**Affiliations:** 1 Key Laboratory of Pollinator Resources Conservation and Utilization of the Upper Yangtze River, Ministry of Agriculture and Rural Affairs, Chongqing Normal University, Chongqing 401331, China; 2 Chongqing Key Laboratory of Vector Control and Utilization, College of Life Sciences, Chongqing Normal University, Chongqing 401331, China

**Keywords:** *Vairimorpha (Nosema) ceranae*, spore wall protein, polar tube proteins, subcellular localization, biological functions

## Abstract

*Vairimorpha (Nosema) ceranae* is a pathogen that affects *Apis mellifera* and *Apis ceranae* Fabricius, capable of spreading within and between honeybee colonies. The spore wall of microsporidia is the initial structure to contact the host cell directly, which may play a crucial role in the infection process. Currently, several spore wall proteins have been identified in microsporidia, but only two spore wall proteins from *V. ceranae* have been characterized. Here, we report the expression and identification of a novel spore wall protein, NcSWP8, with a molecular mass of 21.37 kDa in *V. ceranae*. Subcellular localization analysis revealed that NcSWP8 was localized on the spore wall of *V. ceranae*. Co-immunoprecipitation and Far-Western blotting experiments demonstrated that NcSWP8 could stably interact with polar tube proteins, NcPTP2 and NcPTP3. The antibody blocking assay significantly decreased their infection rate, indicating that NcSWP8 played a significant role in the process of *V. ceranae* infection. These results together suggested that NcSWP8 was a new spore wall protein localized to the spore wall and interacted with the polar tube proteins, playing a crucial role in supporting the formation of the spore wall and potentially affecting the process of infection of *V. ceranae*.

## 1. Introduction

Microsporidia are a class of obligate intracellular parasitic eukaryotes found in various hosts, including vertebrates and invertebrates [1]. The first honeybee microsporidium, *Vairimorpha (Nosema) apis*, was identified in *Apis mellifera* in 1909 [2]. Subsequently, researchers have discovered *Vairimorpha (Nosema) ceranae* from the Eastern honey bees, *Apis ceranae* [3]. It is reported that *V. ceranae* has a tremendous capacity for infection; it can infect both *A. mellifera* and *Apis ceranae* Fabricius [4]. The infection begins in the midgut epithelial cells of honeybees, where *V. ceranae* matures and proliferates extensively [5]. Infected bees suffer from digestive disorders, shortened lifespans, diminished foraging ability, increased vulnerability to bacterial infections, and other issues, resulting in a decline in bee populations and ultimately the collapse of the entire colony [6]. For the past few decades, fumagillin has been one of the most widely used commercial antibiotics available for treating *Vairimorpha (Nosema)* infections [7,8]. While it inhibits the reproduction of *Vairimorpha (Nosema)* in honeybees, it does not eliminate the parasites, resulting in frequent relapses [9]. Reports indicate that fumagillin remains effective against *N. apis*; however, its efficacy against infections caused by *V. ceranae* has been less pronounced [10]. Furthermore, recent studies have shown that the effectiveness of fumagillin is gradually declining [8,11]. Currently, some researchers have innovatively developed methods using herbal supplements to treat *V. ceranae* infections [12]. However, we are still seeking additional medications or supplements that can effectively control *V. ceranae*. Therefore, investigating the pathogenic mechanisms of *V. ceranae* is crucial for discovering new treatments to protect honeybees.

The spore of microsporidia has three basic structures: the thick wall, the plasma membrane, and the unique infection apparatus, which includes a polar tube, a polaroplast, and a posterior vacuole [13]. The spore wall of microsporidia, primarily composed of chitin and protein, serves several important physiological functions. For instance, the robust spore walls enable microsporidia to withstand hostile environments effectively [14]. Additionally, the spore wall constitutes the outermost layer of microsporidia, acting as the initial point of contact with the external environment and host cells during the infection process. Reports indicate that spore wall proteins may play roles in spore adhesion, signal transduction, and other interactions with host cells [15,16,17,18]. Consequently, investigating the composition of the spore wall and the biological functions of spore wall proteins is of great significance.

In recent years, an increasing number of researchers have concentrated on identifying new spore wall proteins in microsporidia and their specific functions [19]. To date, over 20 distinct types of spore wall proteins have been discovered, primarily focusing on *Encephalitozoon* and *Nosema*. The researchers identified EcSWP1, EiSWP1, EiSWP2, EhSWP1a, and EhSWP1b as outer spore wall proteins, while EcEnP1, EcEnP2/EcSWP3, EcCDA, and EiEnP1 were classified as inner spore wall proteins in *Encephalitozoon cuniculi* (Ec), *Encephalitozoon intestinalis* (Ei), and *Encephalitozoon hellem* (Eh) [14,16,17,20,21,22,23,24]. Among these, EcSWP1 was the first spore wall protein of microsporidia to be identified, expressed in significant quantities only after spore maturation [24]. EcEnP1 plays a significant role in regulating the infection process of host cells by interacting with glycosaminoglycans (GAGs) present on the surface of these cells [16]. In the *Nosema*, spore wall proteins of *Nosema bombycis*, such as NbSWP5, NbSWP7, NbSWP9, NbSWP11, NbSWP12, NbSWP16, NbSWP25, NbSWP26, NbSWP30, NbSWP32, and EOB14572, have been identified [25,26,27,28,29,30,31,32,33]. Among these, NbSWP5 can interact with polar tube proteins (PTP2 and PTP3), and treatment with Anti-NbSWP5 can reduce the infection rate of the host [34]. NbSWP9 can also interact with polar tubes, aiding in anchoring and arranging them in an orderly manner [25]. Additionally, the researchers also identified other spore wall proteins such as *Antonospora locustae* SWP2 (AlocSWP2) and *Enterocytozoon hepatopenaei* SWP1 (EHSWP1) [35,36].

Currently, various spore wall proteins of microsporidia have been reported to be closely associated with the formation of the spore wall and the infection, while studies related to *V. ceranae* are seldom reported. Thus, it is essential to systematically identify and analyze the biological functions of the new spore wall proteins of *V. ceranae.* Up to now, only two types of hypothetical spore wall proteins, AAJ76-1400036761 and NcER_100148, have been reported in *V. ceranae* [18,37]. The former interacts with chitin shells and contributes to the construction of spore walls, helping to maintain the complete morphology of spores. The latter may play significant roles in the formation of endospores, coiling and fixation of polar tubes, as well as regulating infection in host cells.

In this study, the expression and localization of NcSWP8 were investigated by gene cloning, prokaryotic expression, indirect immunofluorescence assay (IFA), immunoelectron microscopy (IEM), and immunohistochemical analysis. The findings revealed that NcSWP8 was localized to the spore wall of *V. ceranae*. Furthermore, the biological functions of NcSWP8 were explored using Co-immunoprecipitation (Co-IP), Far-Western blotting, and antibody blocking assays. The results indicated that NcSWP8 played a crucial role in the invasion process of *V. ceranae*.

## 2. Materials and Methods

### 2.1. Sequence Analysis

The *NcSWP8* gene sequence of *V. ceranae* was filtered and downloaded from Microsporidia DB (https://microsporidiadb.org/micro/app, accessed on 9 April 2022) by comparing the homologous sequences of *N. bombycis* spore wall protein (access number: NCER_100151). For further sequence characteristic analysis of NcSWP8, ExPASy (http://us.expasy.org/, accessed on 9 April 2022) was used for molecular weight prediction. Putative signal peptide was predicted using SignalP 5.0 (http://www.cbs.dtu.dk/services/SignalP/, accessed on 9 April 2022). Conserved domains were predicted by PFAM (http://pfam.janelia.org/, accessed on 9 April 2022). Server NetNGlyc (http://www.cbs.dtu.dk/services/NetNGlyc/, accessed on 9 April 2022) and YinOYang (http://www.cbs.dtu.dk/services/YinOYang/, accessed on 9 April 2022) were used to predict N- and O-glycosylation sites, respectively. Phosphorylation site prediction was carried out by NetPhos (http://www.cbs.dtu.dk/services/NetPhos/, accessed on 9 April 2022). Glycosylphosphatidylinositol (GPI) anchor motif was predicted using the online program Big-PI Predictor (http://mendel.imp.ac.at/gpi/gpi_server, accessed on 9 April 2022). The Microsporidia DB online tool was used to perform gene collinearity analysis. Clustal X2.0 software, an online tool, was used to perform multiple sequence alignment analysis of the screened target genes. The phylogenetic tree was constructed with the Neighbor-Joining (NJ) method in MEGA 7.0 software, and the evolutionary tree was evaluated with the parameter setting Bootstrapping with 1000 times.

### 2.2. RNA Extraction, Polymerase Chain Reaction (PCR), Semi-Quantitative Reverse Transcription Polymerase Chain Reaction (sqRT-PCR)

PCR and sqRT-PCR were employed to analyze the transcriptional characteristics of *NcSWP8* in spore-infected host honeybees. Firstly, each of the adults of *A. mellifera* in the infected group were infected with 1 × 10^5^ spores of *V. ceranae*. The honeybees in the control group were treated with an equal amount of ddH_2_O instead of the suspension of spores. Then, the midgut samples of the infected and control groups were collected at 2, 4, 6, and 8 days post-infection (dpi), respectively. Midgut samples were taken from five honeybees in each group, and three parallel experiments were conducted as biological replicates within each group. Total RNA was extracted using TRIzol reagent (Sangon, Shanghai, China) and subsequently reverse transcribed into sscDNA using a reverse transcription kit (TaKaRa, Shanghai, China). RT-PCR was then conducted using primers designed by Premier 5.0, which were specific to the *NcSWP8* gene (*NcSWP8*-F:5′-CGCGGATCCATGGCATCTAAAGAAAACTCA-3′; *NcSWP8*-R:5′-CGCGTCGACTTGAGGATAATCATAATCTGC-3′) and the *V. ceranae Actin* gene (*ACT*-F:5′-AATGGTAGGTATGGGTCAA-3′; *ACT*-R:5′-TTTCTTTCAGCAGGTGTTA-3′). The RT-PCR conditions were as follows: 94 °C for 5 min; 94 °C for 40 s, 54.3 °C or 55 °C for 40 s, 72 °C for 90 s, 30 cycles; 72 °C for 10 min, and the period ends at 4 °C. During amplification, the annealing temperatures for the *V. ceranae* actin gene and NcSWP8 were 54.3 °C and 55 °C, respectively. Subsequently, using sscDNA as a template, multiple PCR amplifications were conducted with the corresponding reference gene primers. The amount of template was adjusted until the expression levels of the reference gene were consistent across all sampling times. This amount of template was then used for subsequent PCR amplification. The ImageJ software version 1.54 was used to detect the optical density value of the strips, and the optical density value of NcSWP8 was compared with that of NcActin to obtain the relative expression level of NcSWP8. A *t*-test was performed to test the significance of differences using GraphPad Prism version 5.0. Differences were considered significant if the *p*-value was <0.05. Data were collected from three independent experiments.

### 2.3. Cloning, Expression, Purification of Recombinant NcSWP8, and Preparation of Anti-NcSWP8 Antiserum

According to the gene sequence of *NcSWP8*, the PCR primers *NCSWP8-F* and *NCSWP8-R* were designed as follows: NcSWP8-F:5′-CGCGGATCCATGGCATCTAAAGAAAACTCA-3′; NcSWP8-R:5′-CGCGTCGACTTGAGGATAATCATAATCTGC-3′. The forward and reverse primers contained *BamH* I and *Sal* I restriction sites, respectively. The *NcSWP8* gene was amplified using the genomic DNA of *V. ceranae* extracted via the CTAB method [38,39] as a template and the specific primer pair mentioned above. The PCR conditions were as follows: 94 °C for 5 min; 94 °C for 40 s, 55 °C for 40 s, 72 °C for 90 s, 30 cycles; 72 °C for 10 min, and the period ends at 4 °C. Subsequently, the amplified products were separated with a 1.5% agarose gel, then the fragments of the right size were ligated into the pMD19-T vector (TaKaRa, Dalian, China) and transformed into competent cells of *Escherichia coli* strain DH5α. After the DNA construct had been verified by sequencing (Sangon Biotech, Shanghai, China), the amplified fragment and the expression vector pET30a (+) were digested with *BamH* I and *Sal* I and ligated to construct the recombinant expression vector NcSWP8-pET30a, which was subsequently transformed into *E. coli* Rosetta (DE3) cells for prokaryotic expression. The recombinant rNcSWP8 was induced at 16 °C for 24 h in the presence of 0.1 mmol/L IPTG and purified with Ni-NTA affinity chromatography (Qiagen, Beijing, China). Subsequently, five mice were immunized with rNcSWP8 mixed with Freund’s adjuvant (1:1; Sigma-Aldrich, St. Louis, MO, USA), and two more mice were injected with phosphate-buffered solution (PBS) as the negative control. Then, they were immunized four times (complete Freund’s adjuvant was used for the first time, and incomplete Freund’s adjuvant was used for the rest), each time with an interval of seven days. Following this, the Anti-NcSWP8 serums and the mouse negative serums were collected and stored at −80 °C for further use in Co-immunoprecipitation (Co-IP), Far-Western blotting, and antibody blocking assay.

### 2.4. Specificity Analysis of Polyclonal Antibody Anti-NcSWP8

Spores (10^9^ spores) were disrupted using 0.4 g glass beads, 5 μL phenylmethanesulfonyl fluoride (PMSF), and 500 μL RAPI cracking solution in the crusher (Next Advance, New York, NY, USA) for 5 min. After centrifugation for 20 min at 4 °C at 12,000 rpm, the supernatant was collected as the total spore protein of V. ceranae. The total protein and rNcSWP8 were separated by 12% SDS-PAGE and transferred to a PVDF membrane. The membranes were blocked with 5% (c) skim milk overnight at 4 °C and then washed with Tris-buffered saline with Tween-20 (TBST). Following three washes, the membranes were treated for 1 h at 37 °C with mouse Anti-NcSWP8 serum (diluted 1:500 in cleaning buffer solution). After washing again, the membranes were reacted with HRP-labeled goat anti-mouse IgG (1:5000 dilution; Sangon Biotech, Shanghai, China) for 1 h at room temperature (RT) and washed 3 times with TBST. Finally, the blot was developed with the TMB color development kit (Beyotime, Shanghai, China).

### 2.5. Subcellular Localization Analysis of NcSWP8

#### 2.5.1. Immunofluorescence Analysis (IFA)

An appropriate number of spores were fixed in 4% (*w*/*v*) paraformaldehyde for 10–15 min at room temperature. After washing with phosphate-buffered solution (PBS), spores were treated with 2% (c) Triton X-100 for 15 min. After rinsing three times with PBS, the spores were blocked with 50 μL of blocking solution (containing 5% (*v*/*v*) BSA and 10% (*w*/*v*) goat serum in PBST) for 1 h at room temperature. The sample was then incubated with mouse Anti-NcSWP8 (1:100) for 1 h at room temperature. The negative control group was incubated with the mouse negative serum. After three washes with PBS, the spores were treated with FITC-conjugated goat anti-mouse IgG (1:1000) for 1 h at room temperature. After washing three times with PBS, the DNA was stained with DAPI for 20 min. An anti-fluorescence quenching agent was then added dropwise after washing three times with PBS, and spores of *V. ceranae* were examined using an Olympus FV1200 laser confocal microscope (OLYMPUS, Tokyo, Japan) after preparation.

#### 2.5.2. Immunoelectron Microscopy Analysis (IEM)

The immunoelectron microscopy analysis was conducted following the method described by Jaroenlak et al. [36]. Briefly, the samples were fixed overnight using the fixing solution (Servicebio, Wuhan, China) at 4 °C. The fixed samples were rinsed three times with 4 °C PBS (pH 7.4), dehydrated with a gradient concentration of alcohol at −20 °C, and subsequently embedded in resin (HEAD BIOTECHNOLOGY, Beijing, China). The sample embedded in the resin was photo-polymerized and then sliced into sections with a thickness of 70–80 nm using a diamond knife (Daitome, Nidau, Switzerland). The slices were attached to a 200-mesh nickel grid (ZHONGJINGKEYI, Beijing, China) and stored at 4 °C. The treated sample was sealed with a 1% BSA/TBS sealing solution and then incubated with Anti-NcSWP8 and 10 nm gold-conjugated goat anti-mouse IgG (Sigma, Virginia Beach, VA, USA), respectively. The slides were washed with TBS and dried in an oven. The sections were then examined using a transmission electron microscope (HITACHI, Tokyo, Japan).

#### 2.5.3. Immunohistochemical Analysis

Paraffin sections of midgut tissue from honeybees infected with *V. ceranae* were prepared following He Yuanli’s procedure [40]. The slices were boiled in 0.01 mol/L citric acid buffer (pH 6.0) for 15–20 min, then cooled to room temperature and rinsed three times with PBS buffer. The sections were subsequently incubated with sheep serum (10% *v*/*v*) at 37 °C for 1 h. After washing with PBS three times, the sample was treated with Anti-NcSWP8 (1:100) and FITC-conjugated goat anti-mouse IgG (1:1000) at 37 °C for 1 h and 1.5 h, respectively. Additionally, negative mouse serum was used as a control. Following three washes with PBS, DNA was stained with DAPI for 20 min. An anti-fluorescence quenching agent was then added dropwise after washing three times with PBS, and slices were examined using an Olympus FV1200 laser confocal microscope (OLYMPUS, Japan) after preparation.

### 2.6. Biological Function Research of NcSWP8

#### 2.6.1. Co-Immunoprecipitation (Co-IP)

During this procedure, 50 μL of Protein A + G Agarose was washed twice with ddH_2_O and then mixed with 200 μg rNcPTP2 or rNcPTP3 (NcPTP2 and NcPTP3 are two polar tube proteins of *V. ceranae* that have been identified by our experimental team), 200 μg rNcSWP8, and 5 μL Anti-NcSWP8. The mixture was incubated at 4 °C for 10 h, and the incubated agaroses were rinsed seven times with PBS buffer. The cleaned agarose was combined with 60 μL of electrophoretic loading buffer and treated in boiling water for 10 min, serving as the treated groups. The same volume of mouse negative serum was used to create the samples for the negative control groups instead of the Anti-NcSWP8 mentioned above. The interacting mixture of rNcPTP2 (or rNcPTP3) and rNcSWP8 was utilized to prepare the samples for the positive control groups. All three groups underwent SDS-PAGE electrophoresis and transmembrane transfer. The membranes were incubated with 1:2000 (*v*/*v*) diluted Anti-NcPTP2 or Anti-NcPTP3 and HRP-labeled goat anti-mouse IgG for 1 h, respectively. After undergoing three rounds of washing, employ ECL for color development. The reverse validation method was performed as described above. In order to ensure the scientific validity of the results, three independent Co-IP experiments were performed.

#### 2.6.2. Far-Western Blotting

Far-Western blotting was employed to further identify the protein–protein interactions between NcSWP8 and the midgut protein of *A. mellifera*. The total midgut protein from healthy *A. mellifera* and rNcSWP8 was separated using 12% Sodium Dodecyl Sulfate–Polyacrylamide Gel Electrophoresis (SDS-PAGE) and transferred to a PVDF membrane. The membranes were blocked for 3 h at room temperature and then washed with TBST. Following several washes, the PVDF membranes were incubated at room temperature for 1 h with dialyzed rNcPTP2 or rNcPTP3, 1:2000 (*v*/*v*) diluted Anti-NcPTP2 or Anti-NcPTP3, and HRP-labeled goat anti-mouse IgG, respectively, and were cleaned with TBST three times after each incubation. After ECL color development, photographs were taken, and the images were saved. Reverse the validation method as above. Three independent experiments were performed to ensure the scientific validity of the results.

#### 2.6.3. Antibody Blocking Assay

Spores (10^9^ spores) were washed three times with PBS buffer. The spores were mixed with Anti-NcSWP8 or negative mouse serum and then incubated in a constant temperature water bath at 28 °C for 2 h. The spores were washed three times with PBS and counted again. Three groups were established in the experiment. The blank control group was fed normally, the negative control group was given *V. ceranae* treated with 1:200 (*v*/*v*) diluted mouse negative serum, and the treated group was fed *V. ceranae* treated with 1:200 (*v*/*v*) diluted Anti-NcSWP8 serum. Artificial feeding was conducted at a dose of 1 × 10^5^ spores per bee. Three parallel experiments were set up in each group, and the survival rate of the bees, along with the number of spores in the infected bees, was recorded. To evaluate the number of spores in infected bees, three midguts were randomly sampled and mixed for grinding. Then, it was resuspended with 500 μL of ddH_2_O, and 20 μL was evenly spread on a glass slide for microscopic observation. The number of spores in any three fields of view (400×) was counted, and the average value was taken to represent the infectious amount of spores (spores per field). Data were collected from three independent experiments, and a *t*-test was performed to test the significance of differences using GraphPad Prism version 5.0. Differences were considered significant if the *p*-value was <0.05.

## 3. Results

### 3.1. Characterization of the Gene Encoding NcSWP8

**Figure 1:**
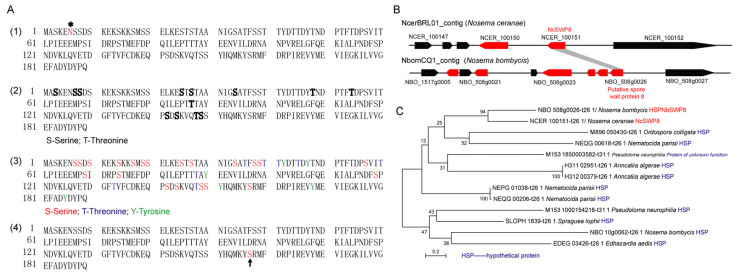
Amino acid sequence and gene collinear analysis of *NcSWP8* and phylogenetic tree of NcSWP8 homologous sequence. (**A**) Amino acid sequence analysis of NcSWP8. (**A1**) Prediction of N-glycosylation site of NcSWP8; “*” represents the N-glycosylation site. (**A2**) Prediction of O-glycosylation site of NcSWP8; bold letters represent the O-glycosylation site. (**A3**) Prediction of phosphorylation site of NcSWP8. (**A4**) Prediction of GPI anchor site of NcSWP8; arrow represents the GPI anchor site. (**B**) Gene collinear analysis of *NcSWP8*. (**C**) Phylogenetic tree of NcSWP8 homologous sequence.

The gene coding for NcSWP8 was 585 bp and encoded a 189-amino-acid protein with a calculated molecular mass of 21.37 kDa and a predicted pI of 4.35. Additionally, the prediction results indicated that the NcSWP8 protein lacked a signal peptide, transmembrane domain, and typical functional domain. According to NetNglyc and YinOYang analysis, NcSWP8 contained one potential N-glycosylation site (asparagine/N at position 6) and thirteen O-glycosylation sites in the protein sequence (Figure 1(A1, A2)). A total of 37 potential phosphorylation modification sites were predicted in the protein, including 21 serine/S sites, 10 threonine/T sites, and 6 tyrosine/Y sites (Figure 1(A3)). The serine/S at position 157 of the protein sequence also has a GPI anchoring site (Figure 1(A4)).

Colinear analysis (Figure 1B) revealed that *NcSWP8* was colinear with the position of the *NBO_508g0026* gene on the NbomCQ1 contig in the *N. bombycis* genome database. The *NBO_508g0026* gene was annotated as the putative spore wall protein 8 (NbSWP8), indicating that the gene locus was conserved in the genomes of *V. ceranae* and *N. bombycis*. Furthermore, the phylogenetic tree (Figure 1C) demonstrated that *NcSWP8* of *V. ceranae* and *NbSWP8* of *N. bombycis* were clustered into one branch, with NcSWP8 and NbSWP8 grouped together, suggesting that they were evolutionarily close and may share conserved biological functions.

**Figure 2:**
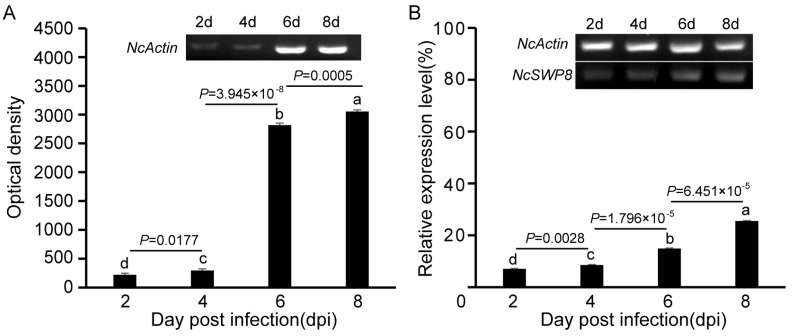
(**A**) Transcription profiles of *NcActin* with the development of infection by *V. ceranae*. (**B**) Transcription profiles of *NcSWP8* at different stages of infection. Data are the mean ± standard errors of three independent replicates, and the letters above the column indicate significant differences between different groups (*p* < 0.05).

The *NcSWP8* transcription was detected by PCR at various intervals following infection. The results in Figure 2A indicated that the amount of the *NcActin* gene gradually increased with the duration of infection. Amplified bands were observed from the second day, confirming that *V. ceranae* had successfully infected *A. mellifera*. The mean optical density (OD) values of the *NcActin* gene were 221, 295, 2791, and 3033 at 2, 4, 6, and 8 days post-infection (dpi), respectively. On the sixth day after infection, the optical density value of the *NcActin* gene significantly increased by approximately 10 times compared to the fourth day. The results indicated that the number of *V. ceranae* obviously increased from 4 dpi to 6 dpi. The successful infection of *V. ceranae* provided a prerequisite for the subsequent analysis of the expression profile of *NcSWP8*. Subsequently, the *NcSWP8* transcription was detected by sqRT-PCR when the number of spores of *V. ceranae* was consistent. The results in Figure 2B showed that amplified bands were obviously detected from the second day. The relative expression levels of *NcSWP8* were 7.1%, 8.6%, 14.9%, and 25.5% at 2, 4, 6, and 8 dpi, respectively, performing a gradual increase as the infection progressed. It suggested that the transcription of *NcSWP8* could be clearly observed when the mature spores reached a certain quantity, and the expression level gradually increased with the duration of the infection.

### 3.2. Expression and Detection of NcSWP8

**Figure 3:**
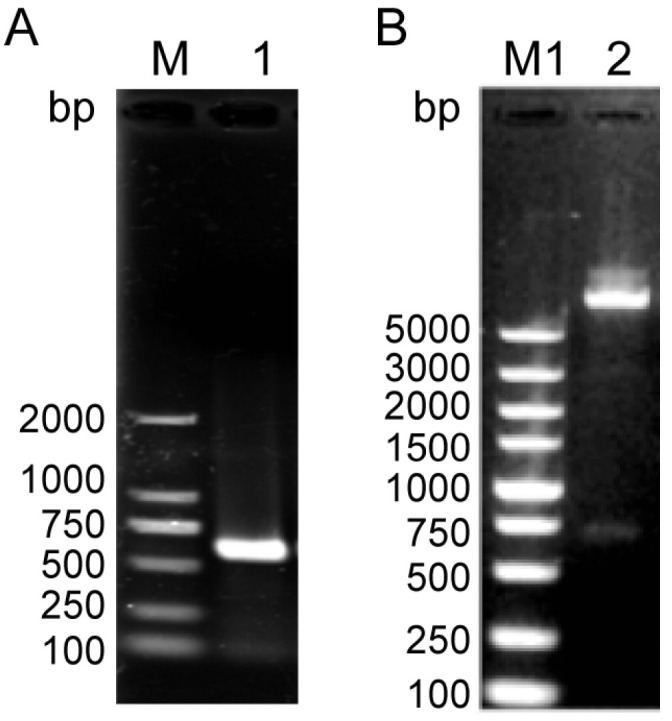
PCR (**A**) and enzyme digestion analysis (**B**) of the NcSWP8-pET30a recombinant plasmid. M: DL2000 Plus Marker; M1: DL5000 Plus Marker; 1: PCR product of NcSWP8-pET30a recombinant plasmid; 2: Double enzyme digestion product of NcSWP8-pET30a recombinant plasmid.

*NcSWP8* was cloned via PCR using specific primers. The target gene was successfully integrated into the pET-30a vector, which was confirmed by PCR and restriction enzyme digestion (Figure 3). The results in Figure 3 showed that a 585 bp fragment, consistent with the molecular weight of the target gene, was obtained from both PCR and restriction enzyme digestion. The sequencing results indicated that the insertion sequence matched the target gene sequence, suggesting that the NcSWP8-pET30a-positive plasmid could be utilized for subsequent recombinant protein generation in *E. coli* cells.

**Figure 4:**
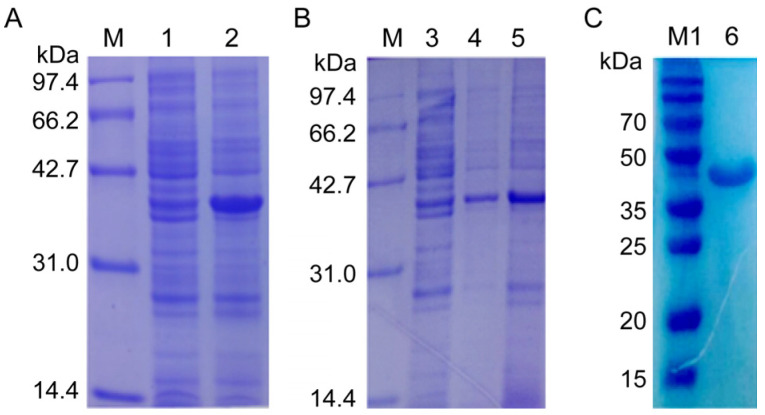
Induced expression (**A**), expression form analysis (**B**), and purification (**C**) of rNcSWP8. M: Protein molecular weight standard; M1: Prestained protein marker; 1: NcSWP8 recombinant protein without IPTG; 2: NcSWP8 recombinant induced by IPT; 3: Uninduced NcSWP8; 4: Precipitation of NcSWP8 after induction; 5: Supernatant of NcSWP8 after induction; 6: Detection of NcSWP8 protein after dialysis.

Then, the recombinant plasmid NcSWP8-pET30a was transformed into *E. coli* Rosetta (DE3) competent cells for overexpression. The results of IPTG-induced expression showed a distinct differential expression band at approximately 40 kDa (Figure 4A), which was consistent with the predicted theoretical molecular weight. The *E. coli* was shattered using ultrasonication and then centrifuged to collect supernatant and precipitate, respectively. The result of SDS-PAGE electrophoresis revealed that the obvious band of the target protein with a molecular mass of approximately 40 kDa was found in both the supernatant and precipitate (Figure 4B), indicating that the recombinant protein rNcSWP8 was expressed in two forms: soluble proteins and inclusion bodies, with a predominance of soluble protein. After collecting a large number of the supernatant, the recombinant protein rNcSWP8 was purified using Ni-NTA affinity chromatography, resulting in a single target protein with the same molecular weight (Figure 4C), confirming that the recombinant protein rNcSWP8 was successfully obtained.

**Figure 5:**
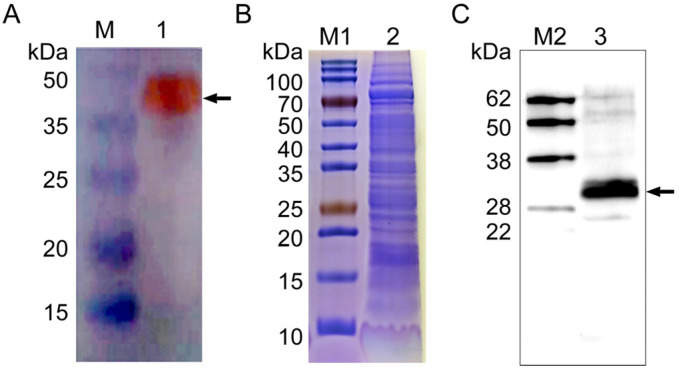
Antibody specificity analysis of Anti-NcSWP8 (**A**), SDS-PAGE analysis of total proteins of *Vairimorpha (Nosema) ceranae* (**B**), and Western blotting analysis of the NcSWP8 in total proteins of *V. ceranae* (**C**). The recombinant protein rNcSWP8 (**A**) and NcSWP8 (**C**) bands are indicated by the black arrow, respectively. M: Protein molecular weight standard; M1: Prestained protein marker; M2: Western blot marker; 1: Recombinant expression protein rNcSWP8; 2: Total proteins of *V. ceranae*; 3: Immunoblotting analysis of the NcSWP8 in total proteins of *V. ceranae* by Anti-NcSWP8.

A polyclonal antibody against rNcSWP8 was generated by immunizing mice with purified recombinant proteins and was utilized in the immunoblotting analysis. A special band about 40 kDa (Figure 5A) was detected using Anti-NcSWP8 as the primary antibody, indicating that Anti-NcSWP8 can specifically recognize the recombinant protein rNcSWP8. Subsequently, total spore proteins were used as antigens and treated with Anti-NcSWP8. The results showed that another special band about 30 kDa (Figure 5B,C) appeared, indicating that NcSWP8 was expressed in the mature spores of *V. ceranae*. This result laid a foundation for the subsequent subcellular localization.

### 3.3. Subcellular Localization of NcSWP8 in V. ceranae

**Figure 6:**
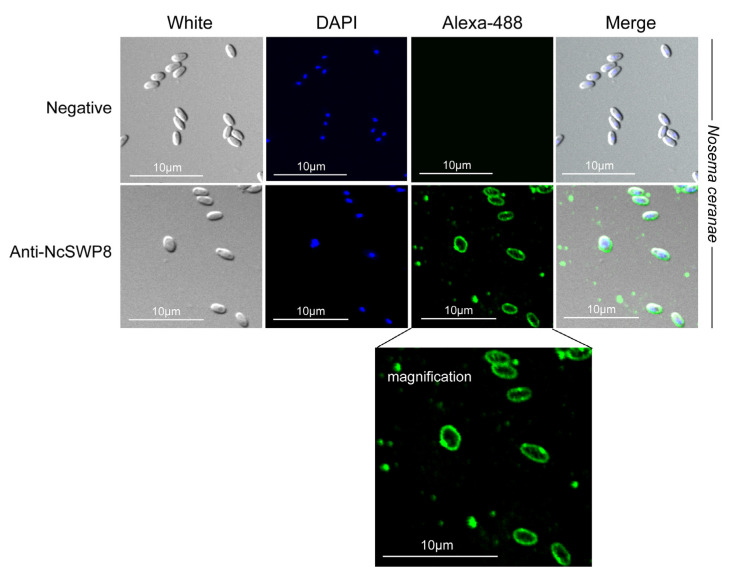
IFA localization of NcSWP8 in mature spores of *V. ceranae*. Immunofluorescence analysis (IFA) reveals the localization of NcSWP8 in the spore wall. Green fluorescence (Alexa-488) indicated the localization of NcSWP8 in mature spores. DAPI was used to stain the nuclei of *V. ceranae* spores (blue fluorescence). Anti-NcSWP8 antibody was used as the primary antibody in the experimental group. Mouse negative serum was employed as the primary antibody in the negative group.

**Figure A1:**
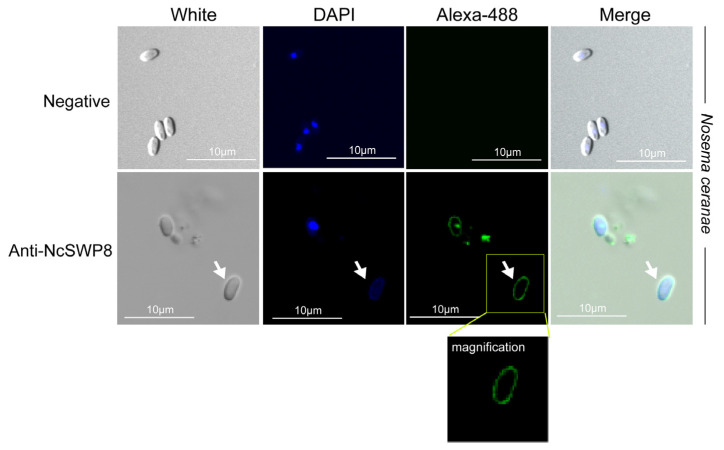
IFA localization of NcSWP8 in the spore coat of *V. ceranae*. Immunofluorescence analysis (IFA) reveals the localization of NcSWP8 in the spore wall. Green fluorescence (Alexa-488) indicates the localization of NcSWP8 in the spore coat. DAPI was used to stain the nuclei of *V. ceranae* spores (blue fluorescence). Anti-NcSWP8 antibody was used as the primary antibody in the experimental group. Mouse negative serum was employed as the primary antibody in the negative group.

When Anti-NcSWP8 was utilized to conduct immunofluorescence analysis (IFA) with purified spores of *V. ceranae*, green fluorescence from the Alexa-488 dye indicated that NcSWP8 was distributed across the spore wall (Figure 6). The clear green fluorescence was also observed on the spore coat of *V. ceranae* (Figure A1). In the negative control group, no green fluorescence was observed (Figure 6), suggesting that the NcSWP8 protein was present on the spore surface, potentially serving as a spore wall protein of *V. ceranae*.

**Figure 7:**
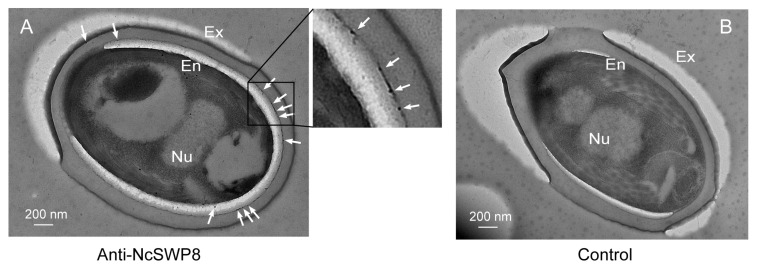
IEM localization of NcSWP8 in mature spores of *V. ceranae*. (**A**) Electron micrographs reveal the localization of NcSWP8. The immunogold particles were indicated with white arrows. (**B**) Negative control probed no signals. Abbreviations: Ex, exospore layer; En, endospore layer; Nu, nucleus.

Further immunoelectron microscopy analysis (IEM) aimed at determining the subcellular localization of NcSWP8 revealed immunogold particles were mainly distributed in the spore wall, including the exospore (Ex) and endospore layers (En) (Figure 7A). Meanwhile, no immunogold particles were detected in the negative control group (Figure 7B). Therefore, these data confirmed that NcSWP8 was a new spore wall protein of *V. ceranae*.

**Figure 8:**
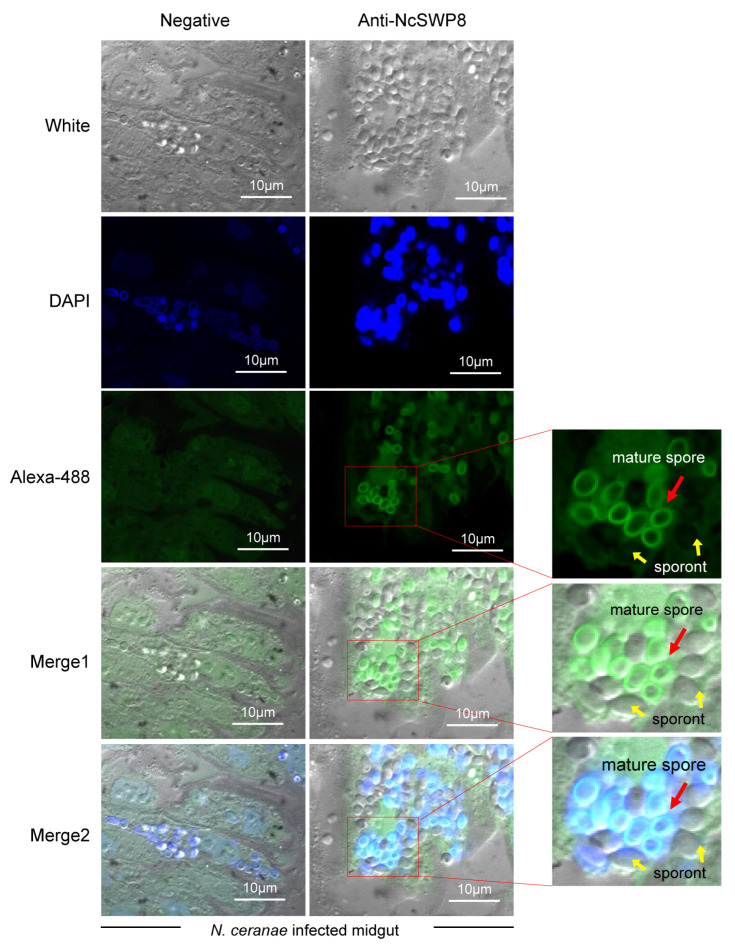
Immunohistochemical analysis of NcSWP8 using Anti-NcSWP8. Green fluorescence (Alexa-488) indicated the localization of NcSWP8 in spores. DAPI was employed to stain the nuclei of *V. ceranae* spores (blue fluorescence). Anti-NcSWP8 antibody was used as the primary antibody in the experimental group. Mouse negative serum was employed as the primary antibody in the negative group. The red arrows showed the mature spores of *V. ceranae*, and the yellow arrows indicated the sporonts of *V. ceranae.*

Next, to further explore the location of NcSWP8 during the proliferation of spores in midgut cells, immunohistochemical analysis was performed. The results in Figure 8 indicated that the midgut cells of honeybees were populated with a significant number of proliferating spores of *V. ceranae*, predominantly mature spores. Clear green fluorescence signals were detected on the surface of mature spores, but not around sporont, suggesting that NcSWP8 may not be involved in the formation of the spore wall during the early proliferation of *V. ceranae*. However, at the mature spore stage, NcSWP8 was highly expressed and fulfilled its corresponding biological functions, aligning with the findings of NcSWP8 transcription characteristics (Figure 2).

### 3.4. Biological Functions of NcSWP8

#### 3.4.1. Interaction Between NcSWP8 and Polar Tube Proteins

**Figure 9:**
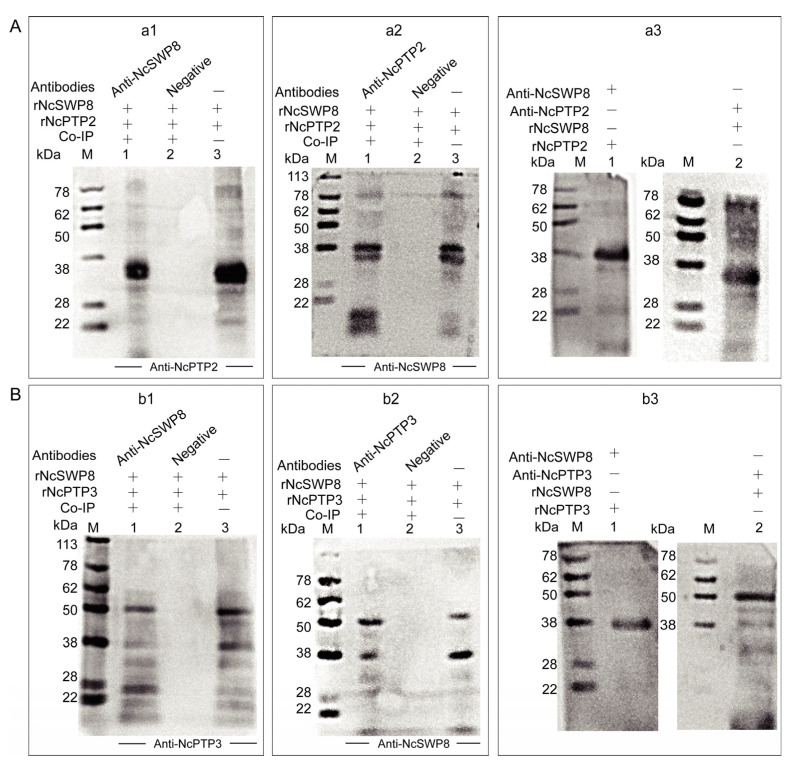
Protein–protein interaction analysis. (**A**) Interaction analysis of rNcPTP2 and rNcSWP8. (**a1**): NcPTP2, NcSWP8, and immunoprecipitated using Anti-NcPTP2 serum. M: protein marker; 1: Anti-NcPTP2 immunoprecipitation results; 2: negative serum immunoprecipitation results; 3: rNcSWP8; (**a2**): NcPTP2, NcSWP8, and immunoprecipitated using Anti-NcSWP8 serum. M: protein marker; 1: Anti-NcSWP8 immunoprecipitation results; 2: negative serum immunoprecipitation results; 3: rNcPTP2. (**a3**): Far-Western blot analysis of rNcSWP8 and rNcPTP2. M: protein marker; 1: rNcSWP8 (incubate the rNcPTP2); 2: rNcPTP2 (incubate the rNcSWP8); (**B**) Interaction analysis of rNcPTP3 and rNcSWP8. (**b1**): NcPTP3, NcSWP8, and immunoprecipitated using Anti-NcPTP3 serum. M: protein marker; 1: Anti-NcPTP3 immunoprecipitation results; 2: negative serum immunoprecipitation results; 3: rNcSWP8; (**b2**): NcPTP3, NcSWP8, and immunoprecipitated using Anti-NcSWP8 serum. M: protein marker; 1: Anti-NcSWP8 immunoprecipitation results; 2: negative serum immunoprecipitation results; 3: rNcPTP3. (**b3**): Far-Western blot analysis of rNcSWP8 and rNcPTP3. M: protein marker; 1: rNcSWP8 (incubate the rNcPTP3); 2: rNcPTP3 (incubate the rNcSWP8).

The aforementioned immunofluorescence assay demonstrated that tiny amounts of black immunogold particles distributed on the polar tube of *V. ceranae*, suggesting that NcSWP8 may interact with certain proteins that constitute the polar tube. To ascertain whether NcSWP8 interacts with polar tube proteins (PTPs), a Co-immunoprecipitation (Co-IP) assay was conducted. The results showed that NcSWP8 co-precipitated with NcPTP3 (Figure 9(a1,a2)) and NcPTP2 (Figure 9(b1,b2)). Compared to the negative serum group, both positive and negative Co-IP assays displayed target bands of the same size as the recombinant protein, suggesting that NcSWP8 could interact with NcPTP2 and NcPTP3. Far-Western blotting was also performed to further confirm the interaction between NcSWP8, NcPTP2, and NcPTP3. The results indicated that Anti-NcSWP8 was able to bind with rNcPTP2 and rNcPTP3, while Anti-NcPTP2 and Anti-NcPTP3 were also able to bind to rNcSWP8 (Figure 9(a3,b3)). The Far-Western blotting experiments further substantiated the Co-IP assay results, indicating that NcSWP8 can interact with NcPTP2 and NcPTP3.

#### 3.4.2. Effect of Anti-NcSWP8 Serum on Infection Ability

**Figure 10:**
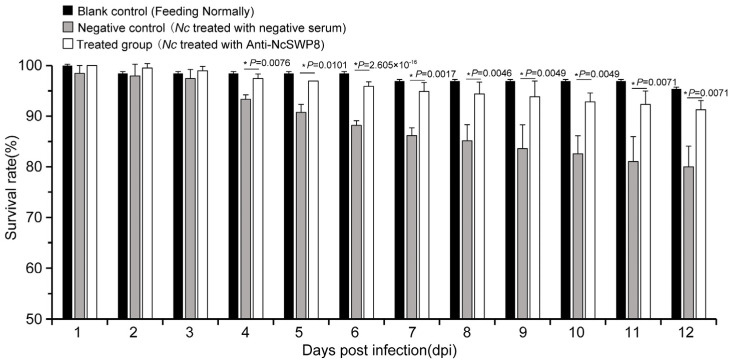
Statistics on the survival rate of honeybees of the antibody-blocking experiment using Anti-NcSWP8. Data are the mean ± standard errors of three independent replicates, and the stars indicate significant differences between different groups (*p* < 0.05).

Many spore wall proteins have a positive effect on the infection of *Vairimorpha (Nosema)*; NcSWP8 is one of them. As shown in Figure 10, the survival rate of the negative control group began to decline significantly on the fourth day, while the survival rate of the treated group decreased slightly, with no significant difference compared to the blank control group, indicating that spores blocked with antibodies are less lethal to bees.

**Figure 11:**
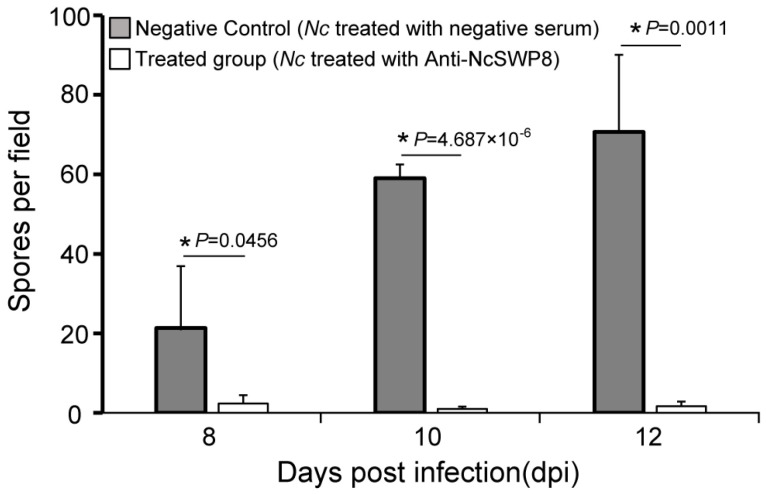
Statistics on the number of spores in the midgut of the honeybees. Data are the mean ± standard errors of three independent replicates, and the stars indicate significant differences between different groups (*p* < 0.05).

As illustrated in Figure 11, there was a significant difference in spore quantity among the two groups: those fed *V. ceranae* treated with negative serum and those fed *V. ceranae* treated with Anti-NcSWP8. In the middle and late stages of bee infection, the number of mature spores in the Anti-NcSWP8-treated group was lower, showing a significant difference from the negative control group. The results indicated that the NcSWP8 on the *V. ceranae* surface was blocked by the antibody, leading to a significant reduction in its pathogenicity and infection capability, suggesting that the NcSWP8 may play a crucial role in the host infection process of *V. ceranae*.

## 4. Discussion

Bioinformatics analysis indicated that NcSWP8 possesses multiple glycosylation sites and a potential GPI anchor site. Additionally, localization analysis showed that it is a spore wall protein, implying that the protein may be anchored to the cell wall through the GPI anchor site or transferred to the cell wall like other fungal GPI proteins [41]. Researchers have previously demonstrated through mutational experiments that both the GPI-anchor site and the N-glycosylation site of SCR76 secreted by *Verticillium dahliae* are essential for SCR76 to effectively suppress the host immune response. These two sites are also critical functional sites for inhibiting host ROS accumulation, electrolyte leakage, and the expression of resistance-related genes triggered by the necrosis-inducing protein produced by *Verticillium dahliae* [42]. BcCFEM1 is associated with the virulence, sporulation, and stress resistance of *Botrytis cinerea*, and the GPI anchor site of BcCFEM1 is closely linked to its performance in these functions [43]. Furthermore, it has also been confirmed in *Candida albicans* that GPI-anchored proteins play a crucial role in the synthesis and repair of the *Candida albicans* cell wall, adaptation to oxidative stress, and invasion of the host [44,45]. In this study, antibody blocking demonstrated that NcSWP8 was significantly related to *V. ceranae* pathogenicity and infectivity. In conclusion, it can be speculated that the GPI anchor site of NcSWP8 may be linked to *V. ceranae* infectivity. Following *V. ceranae* invasion, NcSWP8 may also play a crucial role in spore production, mitigating host oxygen stress, and downregulating host resistance-related gene expression.

The spore wall of microsporidia is composed of spore wall proteins and chitin, which play a crucial role in the resistance of microsporidia to external invasion [14]. Meanwhile, during the process of microsporidia invading host cells, the spore wall serves as the initial structure with which microsporidia contact host cells, thus playing a key role [15,16,17,18]. In this study, Anti-NcSWP8 was able to identify a specific band in the total protein of *V. ceranae*. IFA, IEM, and immunohistochemical analyses demonstrated that NcSWP8 was localized on the spore wall of *V. ceranae*, consistent with the IFA and IEM localization of other spore wall proteins of *V. ceranae*, such as AAJ76-1400036761 [18], NcER_100148 [37], EhSWP3 [46], and NbEOB13250 [47]. Based on these findings, NcSWP8 was recognized as a new spore wall protein of *V. ceranae*.

The general life cycle of microsporidia can be divided into three phases: the infective phase, the proliferative phase, and the spore-forming phase. During the spore-forming phase, chitin and spore wall proteins gradually accumulate along the spore wall [48]. The formation of the spore wall is regarded as a defining characteristic of mature spores in the later stages of microsporidia infection [49]. Consequently, the genes that encode spore wall proteins may either not be expressed or exhibit very low expression levels during the early stages of microsporidia infection. For instance, the expression of *NbSWP26* was only detected during the spore-forming phase, and NbSWP26 was found on the surface of mature spores of *N. bombycis* [50]. However, not all spore wall proteins are exclusively found on mature spores. The expression of *NbSWP12* of *N. bombycis* was observed in both the proliferative and spore-forming phases of microsporidia, with the protein initially localized at the membrane and partially co-localized with the microtubule [51]. Here, the transcription signal of *NcSWP8* could be clearly detected in the middle and late stages (from 6 to 8 dpi) of infection, when the spores reached the spore-forming phase. Additionally, the positioning signal could only be observed on the spore wall of the mature spores, not on the sporont (Figure 8). The results indicated that the expression profile of *NcSWP8* was similar to that of *NbSWP26*, which was primarily conducted during the spore-forming phase. Moreover, NcSWP8 could play a crucial role in constructing the spore wall of mature spores in the later stages of *V. ceranae* infection.

Microsporidia possess a unique infection apparatus, with the polar tube being the most crucial component [13]. Initial studies on the microsporidia infection mechanism indicated that microsporidia spore wall proteins actively contribute to spore germination, and the presence or absence of these proteins directly affects spore germination and, consequently, microsporidia’s capacity to infect the host. Additionally, spore wall proteins may significantly influence the formation and organization of polar tubes. Previous studies have indicated that the spore wall protein NbSWP9 of *N. bombycis* can interact with polar tube proteins, which play a role in linking the polar tube to the spore wall as a scaffold protein [25]. NbSWP5 can influence spore germination through its interaction with tubulin and protect spores from cellular phagocytosis [34]. Similarly, the spore wall protein EOB14572 has been shown to interact with polar tube proteins, and its function is associated with spore germination and spore wall composition [52]. The IEM results for NcSWP8 in this study revealed that some black immunogold particles were also present on the polar tube of *V. ceranae*, indicating that the spore wall protein NcSWP8 may interact with its corresponding polar tube proteins. This study demonstrated that NcSWP8 can indeed interact with NcPTP2 and NcPTP3, speculating that the interaction between NcSWP8 and NcPTPs may facilitate the orderly arrangement and fixation of polar tubes in the spore.

Pathogen surface proteins play a crucial role in the process of host infection and disease, similar to how *Plasmodium* sporozoites, merozoites, and *Toxoplasma gondii* tachyzoites are involved in the infection process [53,54,55,56]. A series of pathogen surface proteins can interact with the receptors of the hosts to mediate invasion in an infection [57]. Spore wall surface proteins are the main components of *Vairimorpha (Nosema)* and represent the first and most direct interaction between the spores and the host, significantly contributing to *Vairimorpha (Nosema)* infection [19]. This study found that spores treated with Anti-NbSWP8 could decrease host mortality compared with the negative control serum, demonstrating that NcSWP8 was involved in the infection process and may participate in the germination of spores. The results regarding NbSWP5 and NbSWP9 in *N. bombycis* were consistent with these findings [25,34]. However, the reasons for this association require further experimentation and investigation. Maybe NcSWP8, which was localized both in the exospore (Ex) and endospore layers (En) of *V. ceranae* spores, like NbSWP5, can respond to stimulation signals during the germination process and help release the polar tube.

## 5. Conclusions

In conclusion, a new spore wall protein, NcSWP8, was identified from *V. ceranae*. NcSWP8 was situated on the spore wall and can interact with NcPTP2 and NcPTP3, which may be linked to the fixation of the polar tube and spore germination. The identification of protein–protein interactions would aid in understanding the invasion mechanism of *V. ceranae*. When the spores were treated with Anti-NcSWP8, the infection rate of *V. ceranae* was significantly reduced. These findings indicate that NcSWP8 played a role in spore invasion of their host cells. This provides valuable data revealing the functions of spore wall proteins.

## Data Availability

The data presented in this study are available on reasonable request from the corresponding author.
